# Melatonin as Modulator for Sulfur and Nitrogen Mustard-Induced Inflammation, Oxidative Stress and DNA Damage: Molecular Therapeutics

**DOI:** 10.3390/antiox12020397

**Published:** 2023-02-06

**Authors:** Eva Ramos, Emilio Gil-Martín, Cristóbal De Los Ríos, Javier Egea, Francisco López-Muñoz, René Pita, Antonio Juberías, Juan J. Torrado, Dolores R. Serrano, Russel J. Reiter, Alejandro Romero

**Affiliations:** 1 Department of Pharmacology and Toxicology, Faculty of Veterinary Medicine, Complutense University of Madrid, 28040 Madrid, Spain; 2 Department of Biochemistry, Genetics and Immunology, Faculty of Biology, University of Vigo, 36310 Vigo, Spain; 3 Health Research Institute, Hospital Universitario de la Princesa, 28006 Madrid, Spain; 4 Departamento de Ciencias Básicas de la Salud, Universidad Rey Juan Carlos, 28922 Alcorcón, Spain; 5 Molecular Neuroinflammation and Neuronal Plasticity Research Laboratory, Hospital Universitario Santa Cristina, Instituto de Investigación Sanitaria-Hospital Universitario de la Princesa, 28006 Madrid, Spain; 6 Faculty of Health, Camilo José Cela University of Madrid (UCJC), 28692 Madrid, Spain; 7 Neuropsychopharmacology Unit, Hospital 12 de Octubre Research Institute, 28041 Madrid, Spain; 8 Chemical Defense Department, Chemical, Biological, Radiological, and Nuclear Defense School, Hoyo de Manzanares, 28240 Madrid, Spain; 9 Dirección de Sanidad Ejército del Aire, Cuartel General Ejército del Aire, 28008 Madrid, Spain; 10 Department of Pharmaceutics and Food Technology, Complutense University of Madrid, 28040 Madrid, Spain; 11 Department of Cell Systems and Anatomy, UT Health, San Antonio, TX 78229, USA

**Keywords:** sulfur and nitrogen mustard, melatonin, oxidative stress, melatonin metabolites, NLRP3 inflammasome, DNA damage

## Abstract

Sulfur and nitrogen mustards, bis(2-chloroethyl)sulfide and tertiary bis(2-chloroethyl) amines, respectively, are vesicant warfare agents with alkylating activity. Moreover, oxidative/nitrosative stress, inflammatory response induction, metalloproteinases activation, DNA damage or calcium disruption are some of the toxicological mechanisms of sulfur and nitrogen mustard-induced injury that affects the cell integrity and function. In this review, we not only propose melatonin as a therapeutic option in order to counteract and modulate several pathways involved in physiopathological mechanisms activated after exposure to mustards, but also for the first time, we predict whether metabolites of melatonin, cyclic-3-hydroxymelatonin, N1-acetyl-N2-formyl-5-methoxykynuramine, and N1-acetyl-5-methoxykynuramine could be capable of exerting a scavenger action and neutralize the toxic damage induced by these blister agents. NLRP3 inflammasome is activated in response to a wide variety of infectious stimuli or cellular stressors, however, although the precise mechanisms leading to activation are not known, mustards are postulated as activators. In this regard, melatonin, through its anti-inflammatory action and NLRP3 inflammasome modulation could exert a protective effect in the pathophysiology and management of sulfur and nitrogen mustard-induced injury. The ability of melatonin to attenuate sulfur and nitrogen mustard-induced toxicity and its high safety profile make melatonin a suitable molecule to be a part of medical countermeasures against blister agents poisoning in the near future.

## 1. Introduction

Sulfur mustards, especially bis(2-chloroethyl) sulfide, known as yperite or mustard gas, and nitrogen mustards, with similar properties to those of their sulfur analogues, being bis(2-chloroethyl)methylamine (HN2) the best known, are the most important blister agents, but not because they are considered a chemical weapon, but because of their use in cancer treatment. These substances are included in the lists of substances subject to CWC verification inspections [1]. In reference to the molecular mechanisms of toxicity induced by blister agents, the following have been reported: the formation of DNA double-strand breaks [2], alkylation of cellular macromolecules [3], activation of Poly ADP ribose polymerase-1 (PARP-1) [4], oxidative stress and generation of reactive oxygen and nitrogen species (RONS) [5], dysregulation of intracellular Ca^+2^ [6], inflammation [7], proteolytic activation [8], and epigenetic modifications [9] have been reported. Unfortunately, immediate total decontamination after mustards exposure is difficult to achieve, and there are not any completely effective and/or efficient antidotes and treatments do not exist. In this complex scenario, the use of a broad-spectrum agent, with a high safety profile and the ability to act on multiple intracellular signaling pathways favoring cell survival, would be a good therapeutic strategy to counteract vesicant agents-induced damage. In this regard, we proposed that melatonin, due to its wide range of biological actions, serving as an indirect antioxidant and free radical scavenger, anti-inflammatory and immunomodulatory agent, or as an epigenetic modulator, and taking into account its low toxicity and high efficacy in reducing oxidative damage and improving human health, should be considered as a strong candidate against exposure and toxicity caused by the most widely used blister agents. 

Melatonin and its metabolites, such as [cyclic-3-hydroxymelatonin (c3OHM), N1-acetyl-N2-formyl-5-methoxykynuramine (AFMK), and N1-acetyl-5-methoxykynuramine (AMK)] exert cell protection against oxidative stress, scavenging and inhibiting the generation of free radicals. Therefore, in this review, we propose mechanisms by which some of these metabolites could mitigate the toxic events elicited by blister agents. Consequently, this protection would further enhance the therapeutic profile of melatonin. 

Another important target activated in response to vesicants is the NLRP3 inflammasome [10], which is associated with a large number of diseases, including inflammatory diseases, metabolic pathologies and carcinogenesis. In this context, melatonin inhibits the NLRP3 inflammasome activity [11] suggesting a new strategy for protecting against blister agents-induced cell death. 

Current understanding of the toxicology associated with exposure to vesicant agents is insufficient to explain, in mechanistic terms, their long-term pathology. In this sense, the effects of variable severity that can occur in the long term after acute intoxication by blister agents, makes us think, once again, on the use of melatonin to modulate epigenetic mechanisms in the clinical treatment of exposed patients.

The purpose of this review article is to highlight the protective role of melatonin and its metabolites in counteracting sulfur and nitrogen mustard-induced damage. 

## 2. Molecular Toxicity Mechanisms of Sulfur and Nitrogen Mustards

The toxicological mechanism of action of vesicants (blistering agents), including the cytotoxic vesicating sulfur and nitrogen mustards, is related to their high reactivity with proteins, DNA, and other cellular components. However, oxidative stress induction (glutathione depletion, lipid peroxidation and reactive oxygen and nitrogen species generation), the activation of poly (ADP-ribose) polymerases (PARPs) (NAD+ depletion and decrease of ATP production), mitochondrial disruption, changes in matrix metalloproteinase-9 (MMP-9) expression, intracellular Ca^+2^ overload, epigenetic mechanisms (long-term toxicity), and programmed cell death, are significant events involved in the molecular toxicity of these vesicating agents [12]. 

The disturbances between reactive oxygen and nitrogen species (RONS) generation and antioxidant defense mechanisms balance, after acute toxicity of sulfur and nitrogen mustards, lead to oxidative stress [13], which is a crucial event of the pathological process causing oxidation of macromolecules, including proteins, nucleic acids and lipids. Currently, all efforts in drug development are directed towards the attenuation of DNA alkylating capacity. However, the generation of RONS and establishment of a scenario of oxidative stress after vesicants exposure, is a more immediate event (hours) than the formation of guanine adducts in contaminated individuals (weeks) [14]. Therefore, in a first approach to reduce sulfur and nitrogen mustards-induced toxicity, the main target would be to counteract oxidative stress.

Production and accumulation of mitochondrial RONS induced by blister agents lead to mitochondrial disruption, mtDNA damage and inhibition of mitochondrial oxidative phosphorylation (OXPHOS) complex inhibition [15]. Recently, Meng et al. [16] have evidenced the presence of a sulfur mustard in the mitochondria of living cells, which indicates that mustards can exert toxicity in cytoplasmic organelles, particularly, dysfunction of mitochondrial dynamics [17]. This increases RONS producing-related enzymes, such as aldehyde oxidase-1 (AOX1), dual oxidases (DUOXs), inducible nitric oxide synthase (iNOS) and thyroid peroxidase (TPO) by reducing both activities of the cytosolic antioxidant enzymes superoxide dismutase (SOD), catalase (CAT) and intercellular glutathione (GSH) [18]. Furthermore, the thioredoxin system and NADPH, critical elements in the protection of all living cells, are necessary for controlling the antioxidant defense system against oxidative stress and protein folding through thiol redox control. In this regard, the nitrogen mustard-derivative mechlorethamine (HN2) inhibits the thioredoxin system inducing overload of RONS and, subsequently, oxidative stress and cellular damage [19]. In addition, RONS can also induce lipid peroxidation (LPO), which leads to LPO–DNA adducts in targeted tissues exposed to mustards [20]. Moreover, an increase in the production of superoxide anions (O_2_^•−^) from mitochondria associated with an uncoupler in the mitochondrial respiratory chain has been observed after mustards exposure [21]. These events compromise the activity of antioxidant defense systems and, therefore, cellular survival.

The most significant effect of mustards in a low dose of exposure is DNA alkylation and cross-linking. PARP-1, in response to DNA damage, uses NAD+ as a substrate to catalyze the covalent binding of poly-ADP-ribose (PAR) and some other nuclear proteins involved in DNA repair. Subsequently, overactivation of PARP-1 and depletion of NAD+-dependent nuclear enzymes by the complex mustards–DNA adducts, without recruitment of the repair system, triggers cell death via necrosis/apoptosis and autophagy [22]. PARP-1 activation is involved in DNA repair; however, overactivation by mustards-induced DNA damage coupled with fast consumption of the NAD+ leads to ATP depletion and cell death [4]. In this respect, intracellular ATP levels act as a cellular sensor to switch the apoptotic or necrotic pathways in response to mustard injury, which may affect the microfilament architecture. In this line, the cytoskeleton organization disruption by exposure to bifunctional alkylating agents sulfur [23] and nitrogen mustards [24], interfere with the mechanisms required for homeostasis maintenance and actin filament cell morphology. It has been proposed that after mustard exposure, PARP-1 is activated and mediates ONOO^−^ induced necrosis; under conditions of low level cell damage, PARP-1 allows DNA repair and cell recovery [25]. This may lead to the delayed toxicity of mustards since cells are able to divide but not be free of damage.

Matrix metalloproteinases (MMPs) are a family of calcium-dependent zinc-binding proteolytic enzymes essential for the remodeling of extracellular matrix (ECM). Gelatinase MMP-9, which promotes proinflammatory pathways and degrades ECM constituents, is up-regulated in lung tissue following sulfur mustard exposure and aggravates the pathogenesis during the progression of a disease [8]. More recently, sulfur mustard was reported to induce corneal structural damage through changes in gene expression of MMPs [26]. Therefore, extracellular matrix proteins and matrix metalloproteinases could be a goal of strategy as a direct therapeutic target against vesicant injury in the ocular tissue. In this context, topical exposure to nitrogen mustard significantly up-regulates MMP9 via MAPK/Akt-AP1 pathway, increasing vesicant-induced skin damage [27]. 

Intracellular calcium [Ca^2+^]_i_ overload is another molecular toxicity mechanism by which vesicants produce cell damage at the acute phase [5]. Ca^+2^ is an essential second messenger for cell homeostasis maintenance and its disturbance provokes triggering cellular pathways that contribute to cytotoxicity. Thus, intracellular [Ca^2+^]_i_ overload after nitrogen mustard exposure triggers autophagy through the TRPV1-Ca^2+^-CaMKKβ-AMPK-ULK1 signaling pathway [28]. Likewise, sulfur mustard is able to increase [Ca^2+^]_i_ mediated by transient receptor potential ankyrin 1 (TRPA1) ion channel/ heat shock 70 kDa protein 6 (HSPA6)-induction [29], which leads to oxidative stress and stimulates cell death. 

Long-term toxicity of mustards affects the quality of life of patients leading to neurobehavioral impairment, cognitive disorders, and severe depression, among others [30]. Furthermore, mustards induce epigenetic modifications without altering the primary DNA nucleotide sequence in cells and tissues exerted by modulators such as histone acetyltransferases (HATs), histone deacetylases (HDACs) and DNA methyltransferases (DNMTs), which play crucial roles in histone acetylation and deacetylation, modulation of chromatin and DNA expression, and cytosine methylation, respectively. Regarding this issue, sulfur mustard induces epigenetic disturbances through DNA methylation and acetylation both in endothelial cells and in vivo skin samples [31]. Dysregulation of HATs and HDACs was also observed after 24 h at low and high dose of sulfur mustard exposure [32]. Regarding the post-transcriptional epigenetic modifications, increasing in serum levels, non-coding microRNAs (miRNAs) were found in humans [33,34] and in rats [35] after sulfur and nitrogen mustard exposure. In this sense, we believe that further clarification of epigenetic mechanisms of mustards may be useful in the development of new therapeutic options.

## 3. Protective Cellular and Molecular Mechanisms of Melatonin against Sulfur and Nitrogen Mustard-Induced Oxidative Stress

As mentioned above, oxidative stress (OS) events have been proposed to play a major role after sulfur and nitrogen mustard exposure. Indeed, after mustard exposure, the induced oxidative environment (ROS, NO and ONOO^−^), including GSH depletion, triggers its detrimental effects cascade [25]. Consequently, a molecule with an effective antioxidant activity would be of therapeutic interest to counteract mustard effects against this key toxic event [36]. Noteworthy, conventional antioxidants cannot remove ONOO^−^. In this regard, melatonin emerges as a promising candidate for a medical countermeasure, with unique features and pleiotropic activities including its well-known antioxidant properties and RONS scavenging potential. Melatonin exerts these effects through several mechanisms [37,38]. Antioxidant therapy is intimately related to inflammation modulation, as NOS inhibitors effectively counteract OS and OS seems to trigger an inflammatory cascade [39].

As a consequence of the described protective effects of melatonin, several authors have studied its antioxidant potency versus mustards, its capacity to detoxify free radicals, as well as other antioxidant properties in rats (Table 1).

The exposure to mustards reduces glutathione peroxidase (GPx) activity which leads to an OS environment. A pre-administration of melatonin in nitrogen mustard-exposed animals protected from GPx loss in a dose-dependent manner [40]. This is quite relevant because while mustards did not diminish significantly SOD levels, other OS markers were altered, i.e., MDA or GPx, which denotes that an OS environment is present in exposed animals. Remarkably, melatonin enhanced SOD activity despite it not previously diminished by mustards [40]. Melatonin showed significant protection for increased oxidized glutathione (GSSG) levels in addition to counteracting the decrease of GSH after nitrogen mustard administration [41]. These results reveal that melatonin’s capacity to neutralize RONS is extensive and complex and, in particular, can directly and immediately combat the short-term damage caused by acute exposure to mustards, as previously reported [14,18].

After nitrogen mustard exposure, RONS overload induces LPO, which is estimated by measuring Malondialdehyde (MDA) or Thiobarbituric acid reactive substance (TBARS) levels, as LPO markers. A study determined that mustard-exposed animals developed strong oxidative stress increasing MDA levels, and melatonin administration significantly diminished MDA levels [40,41], which contributed to membrane protection from LPO. Whereas Pohanka et al. [42] observed that, despite sulfur mustard not seeming to significantly induce changes in plasma levels of LPO compared to the control, when melatonin was previously administered to animals, the LPO marker TBARS was approximately three folds lower, indicating the significative antioxidant activity exerted by melatonin, reducing LPO (Table 1). 

Nitrosative stress markers are just as relevant as oxidative markers. In this regard, there is a large body of evidence that melatonin has an important role in counteracting an increased activity of reactive nitrogen species (RNS) producing related enzymes, as well as selectively inhibiting iNOS [43,44,45]. There is a strong nitrosative stress induction after nitrogen mustard exposure, the expression of iNOS increases generating nitric oxide (NO), which is an unstable nitrogen radical related to ONOO^−^ formation by reacting with superoxide anion (O_2_^•−^). Both NO and ONOO^−^ induce cytotoxicity, modifying membrane lipids, proteins, and DNA covalently [40,44,46,47]; the protection of melatonin at this stage will be extensively discussed in Section 5. Nevertheless, the administration of melatonin seems to reduce nitrosative stress markers (Table 1), measured as the urinary excretion of NO metabolites, nitrite–nitrate (NOx), which is in accordance with the observed inhibition of iNOS activity suppression in treated animals [40,47,48]. Similarly, mice administered intraperitoneally with a single toxic dose of alkylating cyclophosphamide (CP; 200 mg/kg) developed intense oxidative stress in lung homogenates (reducing GSH levels and SOD and CAT antioxidant enzymes), while pre-treatment for seven consecutive days with melatonin (2.5–20 mg/kg) quenched lipid peroxidation and restored normal oxidative parameters [49].

**Table 1:** In vitro and in vivo studies about the protective actions of melatonin against sulfur and nitrogen mustard-induced damage.

Model	Blister Agent (Dose)	Melatonin Dose	Results	Reference
Wistar Rats	Mechlorethamine (0.5 mg/kg)	20 and 40 mg/kg (pretreatment)	↓ LPO (MDA)	[40]
Wistar Rats	Mechlorethamine (0.5 mg/kg)	40 mg/kg (pretreatment)	↑ SOD activity	[40]
Wistar Rats	Mechlorethamine (0.5 mg/kg)	20 and 40 mg/kg (pretreatment)	↑ GPx	[40]
Wistar Rats	Mechlorethamine (0.5 mg/kg)	40 mg/kg (pretreatment)	↓ NOx	[40]
Wistar Rats	Sulfur mustard (20 and 80 mg/kg)	25 and 50 mg/kg (pretreatment)	↓ LPO (TBARS)	[42]
Wistar Rats	Sulfur mustard (20 and 80 mg/kg)	25 and 50 mg/kg (pretreatment)	↑antioxidant power (FRAP)	[42]
Sprague-Dawley rats	Mechlorethamine (3.5 mg/kg)	100 mg/kg/12 h (6 doses post-exposure)	↓ NOx	[47,48]
Swiss mice	Mechlorethamine (10 mg/kg)	250 mg/kg	↓ GSSG	[41]
Swiss mice	Mechlorethamine (5 and 10 mg/kg)	250 mg/kg	↓ LPO (MDA)	[41]
Swiss mice	Mechlorethamine (5 mg/kg)	250 mg/kg	↑ GSH	[41]

Malondialdehyde (MDA), Superoxide dismutase (SOD), Glutathione peroxidase (GPX), Lipid peroxidation (LPO), Nitrosative stress markers nitrite-nitrate (NOx), Thiobarbituric acid reactive substance (TBARS), Ferric-reducing antioxidant power (FRAP), reduced and oxidized glutathione (GSSG), glutathione (GSH),↑ increase, ↓ decrease.

The studies mentioned in this section treated animals with doses of melatonin from 20 mg/kg to 250 mg/kg (Table 1). Undoubtedly, further investigations are needed to establish the effective dose for each of the wide variety of possible treatments as a preventive measure, and for acute or delayed mustard toxicity.

When melatonin antioxidant power has been studied (Table 1), with the ferric-reducing antioxidant power (FRAP) assay in plasma, a significant situation has been found. While untreated melatonin rats did not present changes, the FRAP for Sulfur mustard + melatonin animals were significantly improved, and this increment was significantly higher compared to only melatonin-treated rats. This leads the authors to think that melatonin’s antioxidant power involves other factors and does not only respond to concentration [42]. 

**Figure 1:**
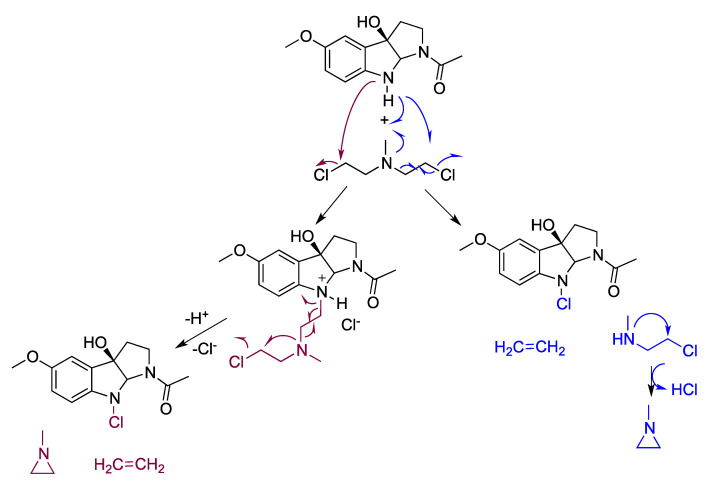
Two proposed mechanisms that may explain the quenching ability of c3OHM on HN2.

Therefore, the described free radical scavenging capacity of melatonin would facilitate the neutralization of sulfur and nitrogen mustards-induced oxidative damage. In this regard, we have previously proposed three mechanisms to achieve melatonin’s protective action: hydrogen-atom transfer (HAT), single electron transfer (SET) and radical adduct formation (RAF) [50,51]. Moreover, melatonin metabolites, c3OHM, AFMK and AMK are also capable of exerting a scavenger action [52]. Therefore, and considering the ability of melatonin metabolites to quench free radicals and thus protect against oxidative damage, we herein propose the possible mechanisms by which some of them could mitigate the toxic events elicited by blister agents such as the nitrogen mustard-2 (HN2) (Figure 1). 

**Figure 2:**
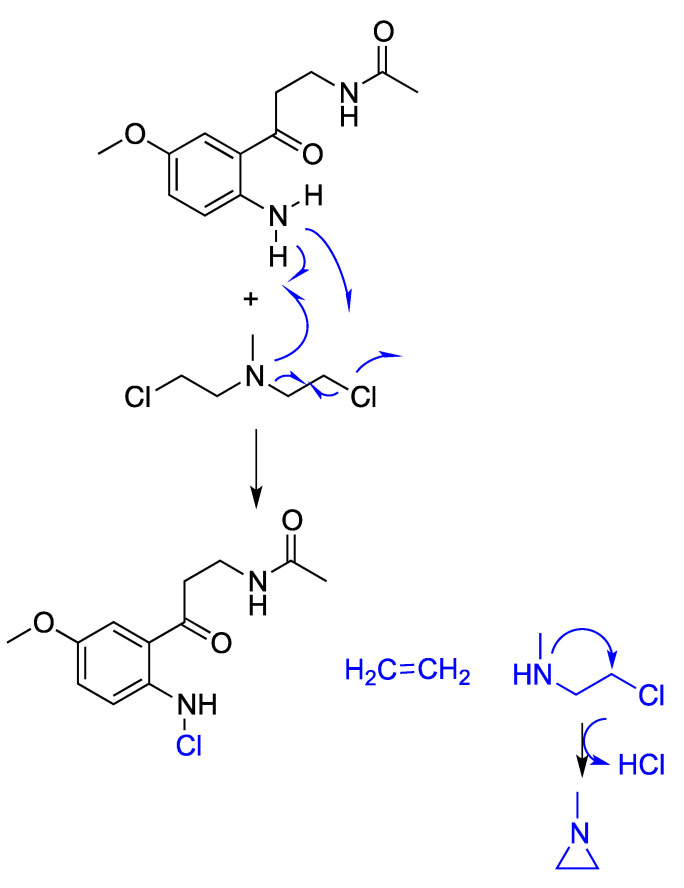
Proposed mechanism of HN2 quenching by the melatonin metabolite AMK.

The melatonin metabolite c3OHM may trap HN2 by nucleophilic substitution, releasing a chloride ion. Otherwise, this chloride ion could leave previously due to an intramolecular nucleophilic attack of the tertiary amine, being then the target for the nucleophilic substitution of the N-methylaziridinium cation. In both cases, the pending alkylating agent may decompose to form ethylene and the less toxic N-methylaziridine, while the chlorine radical is captured by the c3OHM (purple mechanism, Figure 1). Alternatively, this metabolite of melatonin would furnish radical substitution over HN2, taking chlorine radical and provoking the rapid decomposition of this blistering agent to form similar products (Figure 1, blue mechanism). On the other hand, the AFMK metabolite of melatonin does not seem to have a chemical nature to quench this blistering agent. Conversely, the metabolite AMK, despite its soft nucleophilic nature, would develop radical substitution over HN2 to bind chlorine in that step, and thus, inducing the decomposition of this blistering agent in a similar fashion to the c3OHM (Figure 2).

**Figure 3:**
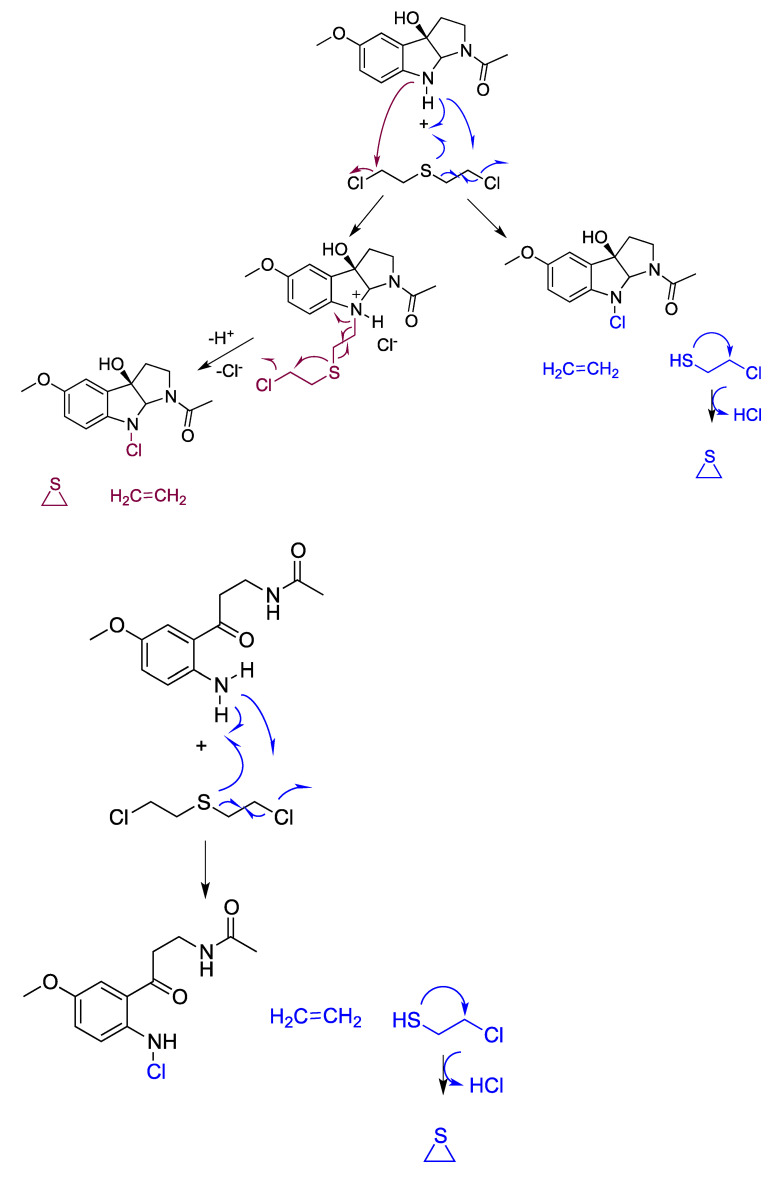
Proposed scavenging actions and mechanisms of breakdown of sulfur mustard by melatonin metabolites.

Similar behavior may be displayed by melatonin metabolites quenching other blistering agents, such as sulfur mustards, which have similar toxic actions. For these mustards, a relevant toxicity mechanism is the formation of alkylthiiranium (namely cyclic alkylsulfonium), which rapidly reacts with water producing thiodiglycol. Consequently, it is essential that these active metabolites of melatonin, c3OHM or AMK, dissipate the sulfur mustard before the alkylthiiranium generation avoiding, thus, this toxic mechanism (Figure 3).

Subsequently, and taking into account the review by Tan et al. [53], the capacity of melatonin to counteract the oxidative environment induced by mustards does not only lie in melatonin as the parent compound, but also in the endless fashion due to the action of many melatonin metabolites, i.e., the antioxidant cascade. Together with its unique features, its metabolite activities may reinforce the potency of melatonin reverting a mustard’s toxic actions compared to other classical antioxidants.

## 4. Sulfur and Nitrogen Mustard-Induced Inflammation: Therapeutic Regulation of the NLRP3 Inflammasome by Melatonin

As previously mentioned, sulfur and nitrogen mustards are bifunctional lipophilic alkylating agents that rapidly penetrate tissues and cells and react with sulfhydryl, carboxyl, and aliphatic amino groups to form stable adducts [54]. This causes oxidative and nitrosative stress, impairs cell function, causes DNA damage, and triggers cell death, apoptosis and autophagy [55,56]. In experimental models and humans, mustard-induced acute and long-term injury is associated with RONS, which are closely associated with mustard-induced inflammation. DNA-damaged cells undergo activation of various signaling pathways, such as PARP, p53 and NF-κB, which play an important role in DNA damage repair, cell cycle arrest and/or inflammation [57]. It has been shown that exposure to mustards increases p53 levels and p53 phosphorylation, mediating early apoptosis and inflammation [58,59]. Apoptotic cell death itself can activate innate immune responses and increase inflammation [60]. Indeed, cell death induced by mustard exposure produces the release of various pro-inflammatory mediators, such as TNF-α, IL-1α or IL-1β, and stimulates the activation of resident macrophages and mast cells, hence activating an immune response. These inflammatory cells also release inflammatory mediators that activate neutrophil extravasation and accumulation at the injured area [61]. After tissue infiltration, neutrophils generate chemotactic signals to attract monocytes and macrophages to the injured area. Depending on the stage of injury, these cells may exhibit a proinflammatory phenotype or aid in wound healing. Tissue repair involves the phagocytosis of apoptotic neutrophils, dead cells and debris at the site of injury and the release of various growth factors that may help promote cell proliferation and extracellular matrix synthesis [62]. However, during prolonged tissue stress, these cells may serve as a source of inflammatory mediators and cytokines that may support further neutrophil infiltration into the injury site [62]. Exposure of rats to vesicants results in marked increases in iNOS, COX-2 and TNFα positive-macrophages in the lung [63]. Moreover, mustards induce the increase of pro-inflammatory cytokines such as IL-6, IL-8, IL-12, and the fibrogenic cytokine transforming growth factor (TGF) β [64]. 

NOD-like receptor family pyrin domain containing 3 (NLRP3) inflammasome is the most widely characterized inflammasome that activates caspase-1 and induces IL-1β release. NLRP3 inflammasome is an important step in innate immune responses. Recently, the NLRP3 inflammasome was shown to play an important role in vesicant-induced cutaneous inflammation [10]. The authors describe that nitrogen mustard activates NLRP3 inflammasome through the SIRT3–SOD2–mtROS signaling pathway. The NLPR3 inflammasome has recently been implicated in the immune pathogenesis of several diseases including cardiac, gastrointestinal, pulmonary, metabolic and neurodegenerative diseases. In this regard, inhibition of NLRP3 inflammasome activation has been proposed as a promising novel therapeutic target for inflammation-related disorders. Therefore, mtROS-dependent activation of the NLRP3 inflammasome may be an excellent target to counteract vesicant exposure-induced damage.

Melatonin pleiotropy effects (free-radical scavenger, antioxidant, cytoprotective, anti-inflammatory, oncostatic, anti-aging, immunomodulatory), includes inhibition of NLRP3 inflammasome. ROS is a main trigger of NLRP3 inflammasome activation [65]. Melatonin reduces ROS production in various in vitro models through its antioxidant effect modifying several antioxidant proteins or its ability to scavenge free radicals. In this sense, melatonin reduces NLRP3 inflammasome activation through Nrf2-mediated ROS scavenging [66,67]. Moreover, melatonin diminishes the levels of TXNIP, suppressing ROS production and NLRP3 activity [68]. NF-κB is a key regulator of the first phase (priming phase) of NLRP3 inflammasome activation. Melatonin prevents NLRP3 inflammasome activation by inhibiting NF-κB signaling induced by LPS administration [69]. It is well-known that autophagy is a negative regulator for NLRP3 activation. Recently, our research group showed that LPS inhibits autophagy and increases NLRP3 protein levels and NLRP3 inflammasome activation. Melatonin reversed LPS-induced cognitive decline, decreased NLRP3 levels and promoted autophagic flux in mice [67]. Additionally, melatonin increased of LC3-II/LC3-I, Atg 5 expression and suppressed NLRP3 inflammasome activation in a model of subarachnoid hemorrhage [70]. Furthermore, the regulatory function of melatonin in NLRP3 inflammasome activation in part occurs through post-transcriptional mechanisms, by altering the expression of miRNAs and long noncoding RNAs [71]. In recent years, different groups have proven the modulatory effect of melatonin on the inflammatory response. Hence, on the one hand the NLRP3 inflammasome plays an important role in vesicant-induced inflammation and, on the other hand, melatonin exerts a strong regulatory effect on NLRP3 inflammasome activation. In view of these evidences, this indoleamine may serve as a good therapeutic candidate against vesicant-induced inflammation.

## 5. Role of Melatonin in Counteracting Sulfur and Nitrogen Mustard-Induced DNA Damage

The poisoning by mustards and the subsequent casualties and incapacitating toxicity produced are dire since none of their principal acute effects, including skin blistering, eye disease and acute respiratory distress [72], has a palliative agent [73]. Furthermore, the prolongation of sequelae over time in a pathological network of systemic involvement [74] puts us in the position of finding antidote-based pleiotropic remedies that address the multiplicity of actions gathered by vesicants. In this regard, the spectrum of signaling deployed by melatonin and its metabolites [75,76], as well as their wide distribution in nuclei and organelles [77,78], are consistent with the countermeasures demanded by mustard-induced dysfunctionalities [79]. Specifically, this indoleamine appears to directly remove DNA damage from physical and chemical aggressors [76]. The melatonin cascade also enables extremely efficient electron transfer [80] to scavenge most RONS [25,53,81,82] and the ONOO^–^ [83,84]. In addition, genomic regulation exerted by melatonin modulates pro-oxidant and antioxidant mechanisms and mitochondrial metabolism [85,86], therefore enhancing cellular responses against DNA alteration and chemical damage [51,87,88,89]. This multidimensional response of melatonin towards the repair of modified sites, the elimination of macromolecule damaging agents and the activation of antioxidative defense gives it proficiency to neutralize mustard pathology. 

Along with lung, eye and skin damage [90], sleep disorders are very common in mustard victims [91] and, in this regard, a cross-sectional study conducted with 100 sulfur mustard-injured Iranians from one-third of victims still suffering from poor sleep, respiratory malfunction and other delayed illness from the Iran–Iraq war (1980–1988), showed reduced nocturnal serum melatonin [92]. Moreover, a randomized, double-blind and placebo-controlled trial study in another cohort of 30 Iranian veterans showed the efficiency of melatonin in improving sleep quality [93]. Consequently, the current state of knowledge nurtured from clinical evidence and the functional biology of this indoleamine suggests that replacement therapy with melatonin is a plausible alternative to manage mustard toxicity.

Early pharmacological cytotoxicity of mustard exposure involves chromosomal DNA-damage by readily direct alkylation of purines (in addition to RNA and other macromolecules) and subsequent cross-linking and single or double DNA-strand breaks [22]. In the case that alkylating positions are found on the same strand, the so-called “limpet junction” hinders the access of vital DNA processing enzymes [94]. Otherwise, interstrand DNA cross-linking prevents the uncoiling and strand separation during replication and transcription and therefore, poses serious side effects when cells activate the genome or undergo division [94]. Either way, extensive DNA and/or protein damage exceeds the cell’s clearance capacity, and the resulting processes activate repair mechanisms, oxidative stress and inflammation pathways [22,95,96]. Eventually, this response to genotoxicity can alert the checkpoint machinery to arrest cell cycle progression and activate apoptosis or necrosis [97,98,99]. In this last respect, cellular destruction accounted after extensive chemical damage and DNA fragmentation drives the clinical expression of mustard toxicity. So, skin samples of old male SKH-1 hairless mice treated with 3.2 mg/mouse nitrogen mustard for 30 min resulted in direct DNA damage, as suggested by both increased phosphorylation at Ser 139 of the histone variant H2A.X (called γH2A.X), which signals double-stranded breaks in DNA, and elevation of the oxidative damage marker 8-oxo-7,8-dihydro-2′-deoxyguanosine [100]. Oxidative stress, inflammation, dead epidermis and keratinization disorders were also observed [27,100]. Noteworthy, genome and expressed RNA comprehensive analyses have revealed that melatonin and its two indolic metabolites modulate multiple signaling pathways in cultured human epidermal keratinocytes, including cell differentiation and other relevant activities (antioxidative, antiaging, antiproliferative, etc) for cell homeostasis [101]. Similar to skin-induced injury, human corneal epithelial cells derived from the outermost and highly vesicant-susceptible ocular layer, once exposed to 100 μM nitrogen mustard for 12 to 48 h showed, among other expressions of cell failure, a dose-dependent increase of γH2A.X, proving the damage inflicted to DNA [102]. In view of the pathological expression described, it should be mentioned that at micromolar concentration, melatonin-receptor signaling activated the p38-dependent phosphorylation of p53 and histone H2AX at Ser139, thus gathering DNA-repairing cascades to prevent accumulation of DNA lesions in normal and cancer cells [103,104]. With regard to lung disease, the human bronchial epithelium line 16HBE exposed up to 24 h to yperite (0.25 and 0.5 mM) or its structural and functional analog mechlorethamine (0.1 and 0.25 mM) suffered from both cell detachment and DNA fragmentation in a time/concentration-dependent manner [105]. Regarding the remediation of this harmful, 100 µM melatonin reduced cell detachment by about half, as well as mustard’s interference with cell proliferation, viability and apoptosis by one third [105].

Other evidence supporting the role of melatonin in maintaining molecular machinery and restoring cellular biosystems, is the reported ability to reduce the incidence of spontaneous and chemically induced carcinogenesis, which also invokes upregulation and activation of DNA repair mechanisms [106,107]. It is important to note in this context that DNA maintenance and tumor suppressors appear to be under circadian control [108,109,110], and also, alterations of melatonin regulators affect circadian genes, cancer etiology and patient survival [111]. Indeed, the reduction in breast cancer incidence among blind women has been linked to improved endogenous melatonin and rhythmicity synchronization compared to light-perceptive counterparts [112]. In keeping with melatonin’s capacity to counteract potentially cancerous lesions, the comet assay and wide-genome gene expression screening have shown that pre-treatment with the indoleamine (1 nM) stimulated a functional response in breast and colon cancer cells to repair DNA strand breaks caused by the carcinogen methyl methanesulfonate [113]. Likewise, melatonin (100 and 400 μM) inhibited the induction of sister chromatid exchange by the antineoplastic and potent alkylating nitrogen mustard-derived melphalan [114]. In the same way, low (0.2 mg/kg) and high (0.4 mg/kg) doses of melatonin reduced the accumulation of DNA adducts instilled by the carcinogen safrole [115,116]. Likewise, the radioprotective potential of melatonin has shown to preclude double-stranded breaks in peripheral lymphocytes caused by ^131^I [117] and overall DNA damage in spleen/cerebral [118], hematopoietic [119] and intestinal cells [120] of irradiated mice. Within the scope of genome protection, melatonin also assists reproductive technology to enhance cell homeostasis and functionality. At micromolar or millimolar concentrations, melatonin durably protected the DNA integrity and biochemical functionality of ram [121,122] and rabbit [123] sperm during cryopreservation, as well as reduced the DNA fragmentation and chromatin dispersion of sperm from asthenoteratozoospermic men [124]. Likewise, by inhibiting phospho-histone H2A.X, melatonin prevented double chain breaks in mouse oocytes during prophase arrest [125,126] and in porcine embryos coming from somatic cell nuclear transfer [127]. In short, these and other published data [128] clearly show the anti-mutational and genome-protective capacities that equip melatonin with aptitudes to mitigate the genotoxicity triggered by mustard intoxication.

In addition, to directly damaging the physical structure of macromolecules, strong sulfur and nitrogen mustard poisoning triggers other acute cytotoxicity mediated by the complex signaling pathways of DNA damage repair, cell death, oxidative stress, apoptosis and inflammation [27,59,129,130]. Indeed, high redox imbalance and nitrooxidative stress, in parallel to the decline of antioxidant mechanisms, are crucial in hurting nitric bases and the deoxyribose backbone of DNA [76], as well as releasing pro-inflammatory factors that affect signaling and collapse membranes [40,131]. However, the specific species responsible for the oxidative pathology induced by mustards have not been identified [132], although there is compelling evidence that reactive nitrogen species play a pivotal role in the respiratory damage induced by sulfur mustard [82]. In this regard, it is known that sulfur and nitrogen mustard cause depletion of glutathione (decreases GSH/GSSG ratio) and antioxidant enzymes, resulting in RONS overproduction [41,133,134]. Subsequently, nitric oxide and superoxide combine into the highly reactive nitrosating ONOO^–^ [135], which cannot be cleared by conventional antioxidants and originates a great part of mustard toxicity [39,72,136,137,138]. Peroxynitrite reacts covalently with all major macromolecules and thiols and stresses cellular biosystems to compromise viability and cause apoptosis or primarily necrosis [139,140,141]. Thus, for example, peroxynitrite disrupts membranes through the oxidation of their structural lipids and inactivates the intermediate metabolism due to the inactivating oxidation of enzymes and ion channels essential for DNA repair and energy production [39,142,143]. Moreover, at very low doses, it attacks DNA (with a small preference for guanine and 2′-deoxyguanosine to form 8-nitroguanine and 4,5-dihydro-5-hydroxy-4-(nitrosooxy)-2′-deoxyguanosine adducts, respectively) and produces single strand breaks [144,145]. 

Despite the relevance of peroxynitrite toxicity, to fully understand mustard pathology [146], it is mandatory to take into account the oxidative damage coming from the hydroxyl radical, which is the most common affecting DNA and also displaying preference towards guanosines [147]. Likewise, female mice given percutaneously with sublethal nitrogen mustard exhibited exacerbation of oxidative stress markers in the liver, and DNA damage, which were antagonized at variable levels by oral melatonin (250 mg/kg), better at low (5 mg/kg) than at high (10 mg/kg) concentration of nitrogen mustard [41]. On the other hand, pinealectomized rats receiving melatonin at darkness (1 mg/kg in the drinking water) for 15 days and a terminal intraperitoneal dose of cyclophosphamide (CP) (20 or 50 mg/kg) showed strongly reduced spontaneous chromosomal abnormalities and oxidative lesions of DNA in their bone marrow cells, as well as upregulated DNA excision repair [148]. Furthermore, Sprague-Dawley rats were treated via transdermal with 3.5 mg/kg of the nitrogen mustard-related compound mechlorethamine (MEC) and 30 min later intraperitoneally with melatonin (100 mg/kg), subsequently repeated five times every 12 h, normalized the increase of TNF-α, IL-1β and iNOS produced by the MEC dosage (Table 1) [47]. Histologically, melatonin significantly reduced the alveolar epithelial damage, inflammation and interalveolar septal thickening observed in MEC-treated animals. An identical experimental schedule allowed melatonin to protect against mustard-induced kidney toxicity [48]. By activating the same defense responses, melatonin has also shown significant evidence of being able to protect DNA in other damaging contexts. Thus, the indoleamine provided genetic protection to primary cortical neurons from a rat model of intracerebral hemorrhage [149], human melanocytes [150] and skin fibroblasts [151] damaged by UVB, as well as murine ovaries exposed to genotoxicity and loss of fertility by cisplatin [152]. Notably, melatonin also alleviated oxidative damage of lipids and DNA in primate liver [153] and peripheral blood lymphocytes of healthy, non-smoking males [154]. Additionally, in vitro and in vivo settings have illuminated the broad potential of melatonin to address the genotoxicity of stressors such as arecoline [155], lead ([156]), formaldehyde [157], the endocrine-disrupting bisphenol A [158] or engineered titanium dioxide nanoparticles [159]. 

Remarkably, the detoxifying function of melatonin from oxidizing reactive species has recently been expanded with the suggestion that indoleamine and six of its metabolites are capable of directly reversing oxidative alterations in DNA by transferring electrons to oxidized guanine sites and hydrogen atoms to the sugar moieties of 2-deoxyguanosines [76]. As the authors themselves express, this hypothesis requires experimental research to be verified and thus enlarges the potential of melatonin from prophylaxis and/or attenuation to the direct reversal of oxidative diseases, including mustard poisoning. In view of this and all the other observations compiled earlier, it can be concluded that the anti-genotoxic and macromolecular integrity capacities of melatonin, together with the reinforcement of antioxidant and anti-inflammatory responses, outline a promising horizon for the indoleamine in therapies against morbidity and mortality associated with mustard intoxication. However, in the effort to unravel new clinical management strategies, more research should focus on the myriad actions of melatonin and its proficiency to mitigate the side-effects of mustards.

## 6. Conclusions

In view of the knowledge summarized herein, we have tried to provide an overview of the main molecular and cellular mechanisms involved in sulfur and nitrogen mustard toxicity, which, however, are still being investigated. In this complex scenario, various cytoprotective agents have been suggested to counteract the toxic effects of vesicants, although none of them protect against all pathophysiological manifestations and have so far provided only partial protection. Because it is endowed with multiple functions and a good safety profile, melatonin has a high relevance in the protection against numerous chemical compounds, among them, the blistering agents. Moreover, melatonin also has a differentiating element with respect to other cytoprotective agents; its own metabolites, c3OHM and AMK, exert cytoprotective actions that potentiate the therapeutic capacity reducing the oxidative damage of vesicants. For this reason, it is necessary to further research the role of this indolamine as a preventive/prophylactic agent in preclinical models, as well as in controlled translational trials. In this context, clinicians are paying increasing attention to melatonin, which could potentially have important applications to ameliorate the alkylating agents-induced side effects. But, for this to happen, randomized clinical studies to translate the therapeutic potential of melatonin to clinical practice are needed.

## Data Availability

Data is contained within the article.
